# Pharmacological Perturbation of Mechanical Contractility Enables Robust Transdifferentiation of Human Fibroblasts into Neurons

**DOI:** 10.1002/advs.202104682

**Published:** 2022-03-03

**Authors:** Zheng‐Quan He, Yu‐Huan Li, Gui‐Hai Feng, Xue‐Wei Yuan, Zong‐Bao Lu, Min Dai, Yan‐Ping Hu, Ying Zhang, Qi Zhou, Wei Li

**Affiliations:** ^1^ State Key Laboratory of Stem Cell and Reproductive Biology, Institute of Zoology Chinese Academy of Sciences Beijing 100101 China; ^2^ Institute for Stem Cell and Regenerative Medicine Chinese Academy of Sciences Beijing 100100 China; ^3^ Beijing Institute for Stem Cell and Regenerative Medicine Beijing 100100 China; ^4^ The First Hospital of Jilin University Changchun Jilin 130021 China; ^5^ University of Chinese Academy of Sciences Beijing 100149 China; ^6^ Key Laboratory of Genetic Network Biology, Institute of Genetics and Developmental Biology Chinese Academy of Sciences Beijing 100101 China

**Keywords:** cytoskeleton, lineage conversion, mechanical modulation, small molecules

## Abstract

Direct cell reprogramming, also called transdifferentiation, is valuable for cell fate studies and regenerative medicine. Current approaches to transdifferentiation are usually achieved by directly targeting the nuclear functions, such as manipulating the lineage‐specific transcriptional factors, microRNAs, and epigenetic modifications. Here, a robust method to convert fibroblasts to neurons through targeting the cytoskeleton followed by exposure to lineage‐specification surroundings is reported. Treatment of human foreskin fibroblasts with a single molecule inhibitor of the actomyosin contraction, can disrupt the cytoskeleton, promote cell softening and nuclear export of YAP/TAZ, and induce a neuron‐like state. These neuron‐like cells can be further converted into mature neurons, while single‐cell RNA‐seq shows the homogeneity of these cells during the induction process. Finally, transcriptomic analysis shows that cytoskeletal disruption collapses the original lineage expression profile and evokes an intermediate state. These findings shed a light on the underestimated role of the cytoskeleton in maintaining cell identity and provide a paradigm for lineage conversion through the regulation of mechanical properties.

## Graphical abstract

The authors find that inhibiting mechanical contraction disrupts fibroblast cytoskeleton, promotes YAP/TAZ nuclear exportation, and induces a neuron‐like state which can further maturation. Transcriptomic analysis shows that the cytoskeletal disruption collapses the original expression profile and evokes an intermediate state for neural transdifferentiation. These findings shed light on the underestimated role of the cytoskeleton in maintaining cell identity.

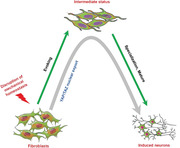

## 1 Introduction

The efficient acquisition of functional cells through lineage conversion is important for regenerative medicine as a means of providing enough cells for replacement therapy in vitro and in situ tissue regeneration in vivo. Obtaining neurons through transdifferentiation holds promise for the treatment of neurodegenerative diseases, which are mainly characterized by the gradual loss of neurons during aging or after damage. The different lineages are stabilized and maintained by lineage‐specific transcription factors (TFs) and gene expression patterns, as well as epigenetic modifications.^[^ ^1^ ^]^ Lineage conversion, such as the transdifferentiation of non‐neuronal cells into neurons, can be achieved by manipulating lineage‐specific transcriptional factors, microRNAs, signaling pathways, and epigenetic modifications.^[^ ^2^, ^3^, ^4^, ^5^, ^6^ ^]^ However, the low transdifferentiation efficiency and the need to simultaneously manipulate multiple genes or compounds limit their therapeutic application.

Mechanical cues can affect genomic organization and transcription^[^ ^7^, ^8^, ^9^ ^]^ and regulate stem cell differentiation.^[^ ^10^, ^11^, ^12^, ^13^ ^]^ Mesenchymal stem cells differentiate along neurogenic, myogenic, and osteogenic lineages when attached to soft, stiffer, and comparatively rigid matrices, respectively.^[^ ^12^ ^]^ Moreover, different somatic cell types are exposed to different physical and mechanical environments in vivo. Neurons and adipocytes stay in soft brain and fat, respectively, whereas muscle cells and osteocytes stay in stiff muscle and even stiffer bone, respectively.^[^ ^13^ ^]^ These observations led us to propose that mechanical manipulation can lead to the fate conversion of somatic cells.

Cells can perceive and respond to extracellular mechanical cues by regulating the cytoskeleton, such as by increasing stress or tension to match the increasing matrix rigidity.^[^ ^14^ ^]^ Nonmuscle myosin II (NMII) and F‐actin filaments together form actomyosin fibers, which are responsible for generating cytoskeletal tension.^[^ ^15^ ^]^ Actomyosin fibers receive mechanical signals from the extracellular matrix through adhesion molecules and transmit them to the nucleus through linker of nucleoskeleton and cytoskeleton (LINC) complexes. The nuclear lamina has a key role in genomic organization and regulation,^[^ ^16^ ^]^ maintaining chromatin compartmentalization by anchoring heterochromatin to the nuclear periphery to preserve transcriptional silence,^[^ ^17^ ^]^ and thereby stabilizing cell identity.^[^ ^18^ ^]^ Additionally, when mechanical signals are transduced to the nucleus, mechano‐responsive transcriptional cofactors such as YAP (encoded by *YAP1*) and TAZ (encoded by *WWTR1*) change their subcellular localization and regulate target gene expression.^[^ ^19^ ^]^ These findings indicate that the disruption of the cytoskeleton may promote fate conversion by regulating chromatin organization and mechanosensitive TFs, thereby promoting new expression patterns.

Here, we showed that disrupting the cytoskeleton using a single compound softens cells, induces YAP/TAZ nuclear export, downregulates the fibroblast transcriptional regulatory network, and evokes a neuron‐like intermediate state which can be effectively re‐specialized to a high‐quality mature neuronal state after the addition of neurogenesis‐promoting factors. These studies shed a light on the underestimated role of the cytoskeleton in maintaining cell identity and provide a paradigm for lineage conversion through the regulation of mechanical properties.

## 2 Results

### 2.1 Inhibition of Actomyosin Contractility by Blebbistatin Disrupts the Fibroblast Cytoskeleton and Induces Cell Softening

**Figure 1:**
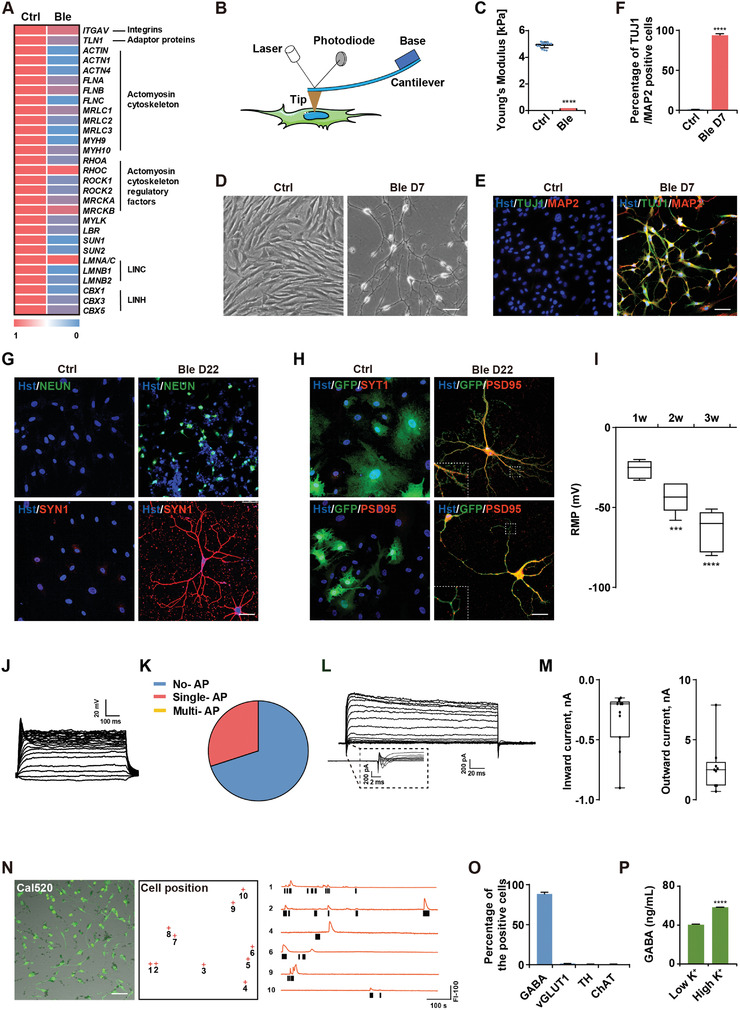
Ble‐mediated inhibition of actomyosin contractility induced cell softening and evoked the neural‐like fate. A) RT‐PCR quantification of the transcriptional level of cytoskeleton‐related genes after Ble treatment. *n* = 3 repeated experiments. B) Atomic force microscopy measurements of cell rigidity showing that C) Ble treatment reduced the cell's Young's modulus, *n* = 3 repeated experiments. D) Human foreskin fibroblast (HFF) morphology after 7 days of DMSO (Ctrl) or (−)‐blebbistatin (Ble) treatment. Scale bar, 50 µm. E) HFFs expressed TUJ1 and MAP2 after 7 days of Ble treatment. Scale bar, 50 µm. F) Quantitation of TUJ1 and MAP2‐positive cells relative to Hoechst‐stained cells after 7 days of Ble treatment. *n* = 10 randomly selected fields from three repeated experiments. G) HFFs expressed NEUN and SYN1 after 22 days of Ble treatment. Scale bar, 50 µm. H) GFP‐labeled Cd‐iNs expressed SYT1 and PSD95 after coculture with mouse astrocytes. Scale bar, 50 µm. I) Statistics of the intrinsic resting membrane potential of HFF‐Cd‐iNs after maturation for 1 (*n* = 8), 2 (*n* = 10), or 3 (*n* = 14) weeks. J) Current‐clamp recordings of Ble‐induced neuron‐like HFFs showing representative single‐spike peaks action potentials. K) The portion of single‐spike peaks in (J) (*n* = 10). L) Representative traces showing Na^+^ (inward) and K^+^ (outward) currents recorded from Ble‐induced neuron‐like HFFs. M) Quantitative evaluation of inward current and outward current in (L). *n* = 11 for inward current, *n* = 9 for outward current. N) Fluorescence image of Cd‐iNs stained with the calcium indicator Cal520; red crosses indicate the cell positions (top). Representative traces of spontaneous calcium transients; black vertical bars indicate the initial time of calcium transients (bottom). O) Quantitation of GABA, vGLUT1, TH, and ChAT‐positive cells relative to Hoechst‐stained cells at day 30. *n* = 10 randomly selected fields from three repeated experiments. P) GABA release by Cd‐iNs measured by UPLC/HRMS under high or low K^+^ conditions. *n* = 3 repeated experiments. Data represent means ± SEM. ^***^ *p* < 0.001, ^****^ *p* < 0.0001 (*t*‐test in (C,F,I,P)).

To study whether inhibition of actomyosin contractility disrupts the fibroblast cytoskeleton, we treated human foreskin fibroblasts (HFFs) with a selective NMII inhibitor, (−)‐blebbistatin (Ble), and subsequently measured the transcriptional levels of genes encoding cytoskeletal components and regulators. We found that the expression of most of these genes was downregulated within 24 h of Ble treatment (**Figure** **1**A). Moreover, the cytoskeletal mechanotransduction network collapsed, as evidenced by the presence of fewer vinculin‐containing well‐matured focal adhesions, vimentin fiber meshworks, and perinuclear parallel F‐actin fibers (Figure S1A,B, Supporting Information). To further verify that the effects of actomyosin contractility disruption are transmitted to the nucleus, we used LMNA/C staining to evaluate the distribution of nuclear lamina, as its apical distribution is responsive to actomyosin cytoskeleton‐related tension.^[^ ^20^ ^]^ Following Ble treatment, the apical polarization of the nuclear lamina was reduced (Figure S1C–E, Supporting Information), while 3D reconstruction of the stained nucleus showed that it was deformed, presenting a low‐stress loose state,^[^ ^21^ ^]^ increased height, and smaller nuclear maximum projected area (Figure S1C,F,G, Supporting Information). We also measured the stiffness of Ble‐treated cells by atomic‐force microscopy (Figure 1B), and found that Ble treatment softened the cells, which showed a decrease in Young's modulus (Figure 1C). Together, these results suggested that Ble‐mediated inhibition of actomyosin contractility impaired fibroblast mechanical homeostasis and led to cell softening.

### 2.2 Disruption of the Cytoskeleton Induces a Neural Fate

To determine the effect of Ble‐mediated cell softening on cell fate conversion, we treated HFFs with Ble in serum‐free medium containing FGF2. Surprisingly, live cell tracking showed that the HFFs underwent acute morphological changes within 24 h of treatment (Movie S1, Supporting Information). After 7 days, the original fusiform fibroblasts presented a typical neuronal morphology rich in axon‐like filaments emanating from the plump cell soma (Figure 1D). To rigorously demonstrate this effects, we exclude neuronal contamination in the initial HFFs (Figure S2A, Supporting Information), and we also confirmed that the Ble derivative, (S)‐(−)‐blebbistatin‐O‐benzoate, could also induce a neuron‐like morphology, but not the Ble enantiomer, (+)‐Ble^[^ ^22^ ^]^ (Figure S2B, Supporting Information). Notably, these neuron like cells not only expressed neuronal markers (Figure 1E; Figure S2C, Supporting Information), but also showed remarkable transdifferentiation efficiency with the purity is over 90% (Figure 1F; Figure S2D, Supporting Information). This showed that Ble could efficiently promote a neuronal morphology along with classic neuronal marker expression. Considering that FGF2 was added in our induction system, to further determine whether Ble alone was sufficient to induce neural fate, we added FGF and Ble separately. The results showed that Ble but not FGF2 was sufficient to induce the induction of neuronal fate from human fibroblasts, which could be significantly enhanced by FGF2 (Figure S3A,B, Supporting Information). Furthermore, NMII activation can be triggered by ROCK kinases.^[^ ^23^ ^]^ To investigate whether inhibiting ROCK kinase, thereby indirectly suppressing actomyosin contractility, could also induce a neural fate, we treated HFFs with two ROCK kinase inhibitors, Y33075 and K‐115. Inhibition of ROCK kinase could also convert HFFs into neuron‐like cells with 81.6% (Y33075) and 53.4% (K‐115) efficiencies, respectively (Figure S4A,B, Supporting Information). Finally, to test whether other cell types could be induced to neurons by this approach, we used mouse astrocytes as initiation cells for transdifferentiation, the results showed that astrocytes could also be efficiently transdifferentiated into neurons that expressed the TUJ1, MAP2, and NEUN and PSD95 (Figure S4C–E, Supporting Information), which suggested that this method might have similar effects on different initiation cells which can respond to the inhibition of cell contraction.

Furthermore, when these neuron‐like cells were further cultured in neuron maturation medium, they expressed the mature neuronal markers NEUN and SYN1 (Figure 1G). To test their synapse‐forming ability, GFP‐labeled HFFs were treated with Ble for 7 days and then co‐cultured with mouse astrocytes for 3 weeks for maturation. The resulting GFP‐positive neuron‐like cells expressed the presynaptic and postsynaptic markers, SYT1 and PSD95 (Figure 1H). Furthermore, their resting membrane potential gradually increased during maturation (Figure 1I), these cells had rarely single action potentials and weak Na^+^/K^+^ currents in whole‐cell patch‐clamp recordings (Figure 1J–M), while a calcium flux assay showed that some also displayed spontaneous calcium signals (Figure 1N). Surprisingly, almost all the induced cells were GABA‐positive (GABA^+^), whereas vGLUT11^+^, TH^+^, or ChAT^+^ neurons were rarely detected, indicative of a uniform GABAergic phenotype (Figure 1O; Figure S5, Supporting Information). Moreover, significant GABA release confirmed their GABAergic identity and capacity to respond to stimuli following depolarization using high‐ and low‐potassium solutions (Figure 1P). These results indicated that, although culturing the cells in conventional maturation medium or coculturing them with mouse astrocytes could improve some maturity indicators, the cells were not fully mature and displayed poor electrophysiological characteristics, suggesting that they were early, immature neurons.

Because reducing actomyosin contraction could induce a neuronal‐like phenotype, we next tested whether this process was also responsive to a broader disruption of mechanical homeostasis using siRNAs (Figure S6, Supporting Information). TUJ1‐positive cells were detected after 7 days of culture in induction medium. *MYH9* and *MYH10* encode two NMII heavy chain subtypes, while *ACTB* encodes one of six actin proteins. Consistent with pharmacological inhibition, knocking down MYH9, MYH10, or ACTB also induced TUJ1 expression, while simultaneous MYH9/MYH10 knockdown had an additive effect (Table S1, Figure S7, Supporting Information). BANF‐ and SUN‐containing LINC complexes interact with both perinuclear actin caps of actomyosin fibers in the cytoplasm and nuclear lamina meshes in the nucleus.^[^ ^24^, ^25^ ^]^ Knocking down BANF and SUN also induced TUJ1‐positive neuron‐like cells (Table S1, Figure S7, Supporting Information). These results demonstrated that disrupting the fibroblast cytoskeleton in different nodes could evoke a neural‐like fate.

### 2.3 Enhancing Lineage Specialization Boosts the Neuronal Maturation Induced by Cytoskeletal Disruption

**Figure 2:**
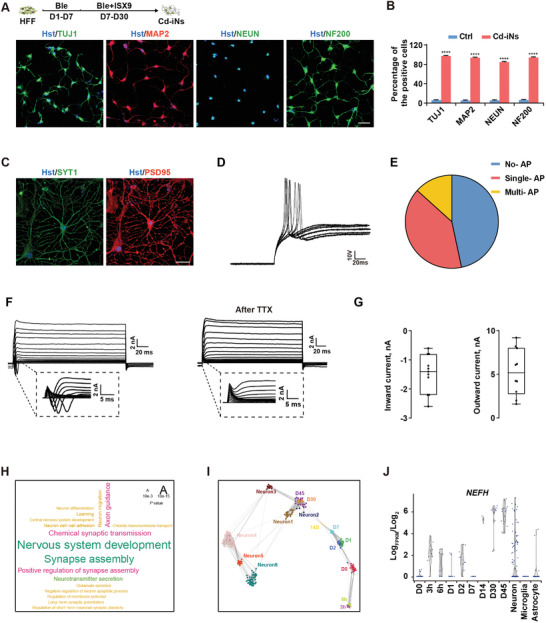
Enhancing lineage specialization promotes neuronal maturation. A) Schematic of the strategy to convert human foreskin fibroblasts (HFFs) into neurons using (−)‐blebbistatin (Ble) and ISX‐9 (top panel). Cd‐iNs displayed complex neuronal morphologies and expressed TUJ1, MAP2, NEUN, and NF200 at day 30 (bottom). Scale bar, 50 µm. B) Quantitation of TUJ1, MAP2, NEUN, and NF200‐positive cells relative to Hoechst‐stained cells at day 30. *n* = 10 randomly selected fields from three repeated experiments. C) Neuron‐like HFF cells expressed SYT1 and PSD95 after Ble and ISX‐9 treatment for 22 days. Scale bar, 50 µm. D) Current‐clamp recordings of Cd‐iNs showed a representative train of action potentials. E) The portion of multi‐spike peaks in (D) (*n* = 15). F) Representative traces showing Na^+^ and K^+^ currents recorded from Cd‐iNs (left panel). Tetrodotoxin (10 µm) treatment inhibited voltage‐dependent sodium currents (right panel). G) Quantitative evaluation of inward current and outward current in (F). *n* = 11 for inward current, *n* = 10 for outward current. H) Gene ontology analysis of genes upregulated in Cd‐iNs (D30/D45). The font size represents *p‐*values. I) Principal component analysis of single‐cell transcriptomes from day 0 (D0), 3 and 6 h, D1, D2, D7, D14, D30, and D45 Cd‐iNs and human primary neurons (Neurons 1–6). J) Violin plot showing RNA‐seq‐derived distribution of the neuronal marker *NEFH* during transdifferentiation across the indicated time points. Data represent means ± SEM. ^***^ *p* < 0.001, ^****^ *p* < 0.0001 (*t*‐test in (B)).

To enhance the maturity of the converted neurons, we used isoxazole‐9 (ISX‐9), a molecule known to promote neurogenesis in adult neural stem cells.^[^ ^26^ ^]^ HFFs were first cultured with Ble in neural induction medium for 7 days, and then in neural maturation medium with Ble and ISX‐9 for 23 days, after which their characteristics were assessed at day 30 (**Figure** **2**A). Following a further 3 weeks of maturation, these neuron‐like HFFs not only expressed a panel of typical neuronal markers, including TUJ1, MAP2, and NEUN (Figure 2A,B; Figure S8, Supporting Information), but also displayed improved synaptogenesis and electrophysiological properties, including evoked action potentials and outward K current and inward Na current and the inward Na current could be blocked by tetrodotoxin (TTX) (Figure 2C,G). We called these cytoskeletal disruption‐induced neurons as Cd‐iNs. Transcriptomic profiling using bulk RNA‐seq demonstrated that the genes upregulated in Cd‐iNs at days 30 and 45 were enriched in nervous system development and neuronal activity (Figure 2H). To ascertain the purity and quality of the Cd‐iNs, single cells were selected at different time points during transdifferentiation for RNA‐seq using publicly available single‐cell transcriptome data for human primary neurons as control.^[^ ^27^ ^]^ Cells at the same time points were highly homogeneous, and different clusters were distinct from each other. Notably, Cd‐iNs at days 30 and 45 fell into two categories of primary neurons (Figure 2I). As an example, NEFH, a typical neuronal marker gene coding neurofilament heavy polypeptide, was consistently highly expressed in Cd‐iNs at day 30 and 45 (Figure 2J).

### 2.4 YAP/TAZ Nuclear Export Induced by the Disruption of the Cytoskeleton is Necessary for Neural Fate Conversion

**Figure 3:**
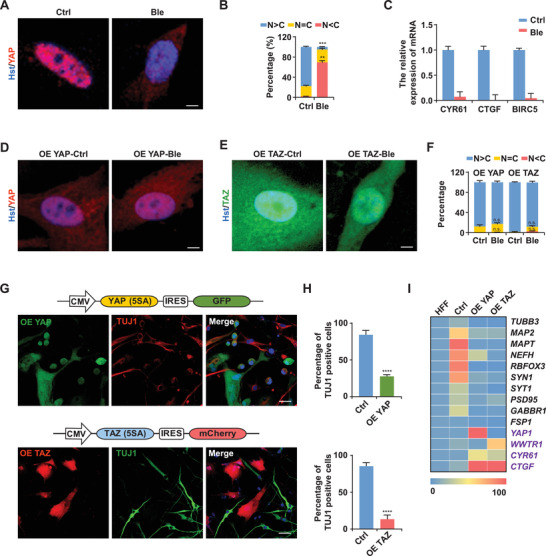
YAP/TAZ nuclear export in response to cell softening is necessary for neuronal conversion. A) Immunocytochemistry of YAP subcellular localization in (−)‐blebbistatin (Ble)‐treated human foreskin fibroblasts (HFFs). Scale bar, 50 µm. B) Statistics for (A). *n* = 3 repeated experiments. C) RT‐PCR quantification of YAP target gene expression (*CRY61*, *CTGF*, and *BIRC5*) in Ble‐treated HFFs. *n* = 3 repeated experiments. D,E) Immunocytochemistry of YAP or TAZ subcellular localization in Ble‐treated HFFs overexpressing YAP or TAZ. Scale bar, 50 µm. F) Statistics for (D,E). *n* = 3 repeated experiments. G) Immunofluorescent staining of TUJ1 in Ble‐treated HFFs with overexpressing YAP (green) and TAZ (red) after 7 days, respectively. Scale bar, 50 µm. H) Quantitation of TUJ1‐positive rate in (G). The “OE YAP” (“OE TAZ”) refers to the ratio of TUJ1 expression of GFP (mCherry) positive cells, while the “Ctrl” refers to the rate of TUJ1 expression of GFP (mCherry) negative cells, respectively. *n* = 10 randomly selected fields from three repeated experiments. I) RT‐PCR quantification of neuronal markers and YAP target genes in wild‐type HFFs and Cd‐iNs derived from control, YAP‐overexpressing, or TAZ‐overexpressing HFFs. Data represent means ± SEM. ^**^ *p* < 0.01, ^***^ *p* < 0.001, and ^***^ *p* < 0.0001 (*t*‐test in (B,F,H)).

In response to mechanical stimuli, YAP/TAZ preferentially accumulate in the nucleus and are activated. As expected, immunofluorescence staining revealed that YAP/TAZ were predominantly localized to the nucleus in HFFs, whereas Ble treatment significantly induced YAP/TAZ nuclear export, which was consistent with the primary neurons with YAP/TAZ mainly localization in the cytoplasm (**Figure** **3**A,B; Figure S9A,B, Supporting Information). Accordingly, three YAP/TAZ‐regulated genes, *CYR61*, *CTGF*, and *BIRC5*, were downregulated after Ble treatment (Figure 3C). To investigate the effect of YAP/TAZ nuclear export on neural fate conversion, we overexpressed the YAP(5SA) and TAZ(5SA), the constitutive nuclear localization mutants for YAP and TAZ, respectively, which the phosphorylation sites of these mutant forms are mutated to alanine, and cannot exit the nucleus^[^ ^28^ ^]^ (Figure 3D). Both YAP(5SA) and TAZ(5SA) overexpression inhibited Ble‐induced YAP nuclear export (Figure 3D,F) and induction of neural fate, as indicated by TUJ1 staining (Figure 3G,H). Additionally, these effects could not be rescued by ISX‐9 treatment, where neuronal genes were downregulated, whereas *YAP1*, *WWTR1* (TAZ), *CYR61*, and *CTGF* were upregulated in YAP(5SA)‐ and TAZ(5SA)‐expressing HFFs compared with wild‐type controls (Figure 3I). Finally, we detected that pharmacological inhibition of TEAD1 by Verteporfin, the YAP/TAZ transcriptional partner, also led to the induction of neuronal fate (Figure S10A,B, Supporting Information). These results suggested that YAP/TAZ nuclear export in response to mechanical cues is necessary for neuronal conversion.

### 2.5 The Landscape of the HFFs‐to‐Cd‐iNs Reprogramming Path

**Figure 4:**
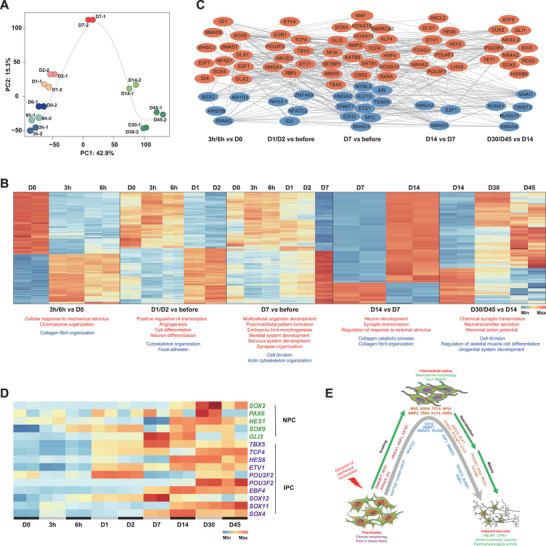
Reconstruction of the human foreskin fibroblast (HFF)‐to‐neuron conversion path. A) Principal component analysis of bulk RNA‐seq transcriptomes from day 0 (D0), 3 h, 6 h, D1, D2, D7, D14, D30, and D45 showing that the reprogramming path resembles an uphill–downhill process. *n* = 2 biological replicates per time point. B) Heat maps (top) and functional analysis (bottom) of differential gene expression at different time points during reprogramming. Red and blue below the marked GO terms and KEGG pathways entries represent significantly upregulated and downregulated genes, respectively. C) Transcription factor networks for different stages of Cd‐iN cell lineage progression. Red and blue nodes indicate upregulation and downregulation at a given stage, respectively. D) The expression of selected genes (rows) marking neural progenitor cells (NPCs) and intermediate progenitor cells (IPCs) are shown during reprogramming. E) Model of neuronal conversion mediated through the disruption of mechanical homeostasis in fibroblasts.

To better understand the mechanism underlying the conversion of HFFs into Cd‐iNs, we reconstructed the associated reprogramming path. First, we performed bulk RNA‐seq for HFFs at day 0 (D0; before induction), 3 and 6 h, and D1, D2, D14, D30, and D45 post‐induction. Principal component analysis (PCA) showed that the global transcriptional level underwent a gradual transition from HFFs to Cd‐iNs along the PC1 dimension (42.9%) (**Figure** **4**A). Notably, along the PC2 dimension (15.3%), the reprogramming process was distinctly divided into two stages, uphill and downhill, and the turning point emerged at D7 when ISX‐9 was added (Figure 4A).

To clarify the major events occurring at each stage of reprogramming, we analyzed the bulk RNA‐seq data of each stage in detail. The results show a heat map of the differentially expressed genes (DEGs) between each time point and the previous ones (Figure 4B). In the first stage (3 and 6 h), genes involved in cellular response to mechanical stimulus (*ITGA2, COL1A1, SOX9*, and *BMP6*) and chromosome organization (*CDCA8, CENPW, GEM, PTTG1*, and *RAD54L*) were upregulated. Concomitantly, genes associated with collagen fibril organization (*FMOD, ADAMTS14, LUM*, and *TGFB2*) were downregulated (Figure 4B, 3/6 h vs D0). This indicated that Ble induced an immediate response in the early reprogramming process. The next stage was associated with cell fate initiation, in which genes involved in positive regulation of transcription, cell differentiation, angiogenesis, and neuron differentiation (*BMP4, NBL1, ZEB1*, and *TCF4*) were upregulated, whereas genes involved in cytoskeleton organization (*ARC, NEDD9, CCN2, TPM1*, and *THY1*) and focal adhesion (*COL4A1, FLT1, PDGFA, COL5A3, FLNC*, and *MYL9*) were downregulated (Figure 4B, D1/2 vs before). Interestingly, an embryonic‐like gene expression profile appeared at D7, characterized by the upregulation of genes involved in multiple developmental programs, including multicellular organism development, proximal/distal pattern formation, embryonic limb morphogenesis, skeletal system development, nervous system development (*NDN, FGF13, JAG1, SIM1, ZNF423*, and *STMN3*), and synapse organization (*ELFN1, NLGN4Y, NLGN2, NLGN3*, and *AGRN*) (Figure 4B, D7 vs before). These results suggested that fibroblast cytoskeleton disruption altered fibroblastic fate and established new, intermediate transcriptional statuses for the development of new lineages, including neural. When the intermediate‐state cells were transferred to an optimized ISX‐9‐containing neuron maturation medium,^[^ ^26^, ^29^ ^]^ genes involved in neuron development and activity (*GDNF, FOXG1, POU4F2, GAP43, KCNC4, GABRB3, SYT5*, and *SYN1*) were upregulated (Figure 4B, D14 vs D7). This specialization phase was followed by a maturation stage in which genes involved in chemical synaptic transmission and neuronal action potential were upregulated, whereas genes involved in regulating skeletal muscle tissue development were downregulated (Figure 4B, D30/D45 vs D14). These results suggested that the intermediate‐state cells could be further specialized into mature neurons.

To further elucidate how destabilizing the cytoskeleton promotes lineage conversion, we constructed a TF network across all the reprogramming stages. The expression of SMAD6/7, which function as inhibitors of BMP and TGF‐*β*/activin signaling,^[^ ^30^ ^]^ and DLX1/2, which promote GABAergic neurogenesis,^[^ ^31^ ^]^ was upregulated as an immediate‐early response to cytoskeletal destabilization following Ble treatment (Figure 4C, 3/6 h vs before). Moreover, some genes involved in the regulation of chromatin organization, such as *WHSC1* ^[^ ^32^ ^]^ and *SATB1*;^[^ ^33^, ^34^ ^]^ promotion of chromatin decondensation and gene expression, such as *HMGA1*;^[^ ^35^ ^]^ and the positive regulation of histone acetylation, such as *KAT6B*,^[^ ^36^ ^]^ were upregulated (Figure 4C, 3/6 h vs D0, D1/2 vs before), whereas genes encoding inhibitors of epigenetic regulation, such as DNMT1 and EZH2, were downregulated at D7 (Figure 4C, D7 vs before). Several genes (*ID1, SOX9, TCF4, ETV1, SOX4*, and *MAF*) were shared with ASCL1‐mediated neuronal transdifferentiation^[^ ^37^ ^]^ (Figure 4C, 3/6 h, D1/D2 vs before, D7).

To verify whether our reprogramming process involved the classic neural progenitor cell (NPC) state, we detected NPC‐related genes across the reprogramming process and found that several (*HES1, SOX9*, and *GLI3*) were expressed during reprogramming. The canonical NPC markers, SOX2 and PAX6, were also markedly transiently upregulated at D30 (Figure 4D). These data suggested that the cells might have gone through a transient NPC stage, although we could not establish NPCs from this process.

## 3 Discussion

Lineage conversion between different cell types induced through the regulation of cell identity‐specific TFs and/or epigenetic modifications have been well described.^[^ ^2^, ^3^, ^4^, ^5^, ^6^ ^]^ However, whether the regulation of cytoskeletal architecture affects the conversion between cell types remains unknown. Here, we efficiently induced a neuron‐like state through Ble‐induced cytoskeletal disruption. Although these intermediate cells expressed mature neuronal markers (NEUN, SYN1), they exhibited weak Na^+^/K^+^ current activity when depolarized after further culture. We therefore speculated that neuronal specialization must be strengthened to achieve neuronal commitment. Consistent with this, the addition of a neurogenic compound, ISX‐9, markedly improved the quality of induced neurons, which displayed a more complex neuronal morphology, a transcriptional level more similar to that of primary neurons, and improved electrophysiological activity. Moreover, single‐cell transcriptomic analysis confirmed the high homogeneity of these induced neurons. Compared with previous reports,^[^ ^5^, ^6^ ^]^ our neuronal reprogramming process was simpler, consisting of a single mechanical disturbance and one neurogenic compound, demonstrating that transdifferentiation toward a neural fate could be efficiently achieved through cytoskeletal disruption.

We investigated how cytoskeletal regulation could trigger the fibroblast‐to‐neuron lineage conversion. YAP/TAZ are sensors of mechanical cues and mainly accumulate in the nuclei of fibroblasts when they are grown on stiff matrices in vitro and help to maintain fibroblast functions, including activation, contraction, matrix synthesis, and proliferation.^[^ ^38^ ^]^ Here, Ble treatment induced YAP/TAZ nuclear export and the downregulation of YAP/TAZ target genes^[^ ^17^, ^18^ ^]^ (Figure 3A,C), suggesting that suppressing cell contractility may inhibit fibroblast function by directly regulating YAP/TAZ and their target genes. In our study, SMAD6/7, endogenous TGF‐*β*/BMP pathway inhibitors,^[^ ^39^ ^]^ as well as DLX1 and DLX2, two TFs that promote GABAergic neuron fate commitment,^[^ ^31^ ^]^ were upregulated during the early stage of Ble treatment, which may provide a guiding environment for neuralization. This suggested that SMAD6/7 are upregulated in response to the cell softening and YAP/TAZ nuclear export (Figures 1C,3A,4E), consistent with previous reports that mechanical stretch can inhibit SMAD6 expression^[^ ^40^ ^]^ and the downregulation of YAP/TAZ activity can induce SMAD7 expression.^[^ ^41^ ^]^ Moreover, several genes involved in regulating chromatin architecture and organization (*WHSC1, HMGA1, RBPJ*, and *SATB1*) were upregulated, while those encoding inhibitors of epigenetic regulation (*DNMT1*, *EZH2*), were downregulated, which may boost the transcriptional reprogramming (Figure 4E). This indicates the existence of a clear framework for lineage conversion mediated through cytoskeletal manipulation. First, changing the mechanical architecture helped to disrupt the fibroblast transcriptional regulatory network. Second, the increased SMAD6/7 expression created an environment conducive to neuralization. Third, regulators of chromatin organization and epigenetic modifications may further have promoted the transdifferentiation process. Finally, these responses together evoked an intermediate state characterized by a disrupted fibroblast transcriptional regulatory network and the emergence of an embryonic‐like gene program, including neurodevelopmental. Moreover, the intermediate state could be effectively specialized to high‐quality and homogeneous neurons after maturation by the addition of a neurogenic factor. Our results suggested that the cytoskeleton may serve as a third dimension to maintain cellular identity, in addition to TFs and epigenetic modifications.

In summary, we used disruption of the cytoskeletal architecture to induce human fibroblast‐to‐neuron lineage conversion. We also report a novel and simple pharmacological transdifferentiation strategy for neurons based on Ble‐mediated cytoskeleton disruption combined with an optimized maturation process in vitro. However, several interesting questions remain to be investigated, such as whether single‐factor mechanical disturbance (e.g., Ble treatment) is sufficient to achieve lineage conversion in vivo in the presence of suitable tissue niches for specialization and maturation. Whether other lineages could be obtained through a similar mechanism that reported here. Considering the variations of cytoskeleton structures among different cell types, such as neurons and adipocytes have less actomyosin cytoskeleton while muscle cells have abundant actomyosin cytoskeleton. Therefore, similar to the neural transdifferentiation, the transdifferentiation of adipocytes may only need to destroy fibroblast actomyosin cytoskeleton in the environment that favors mesoderm development. However, for muscle cell transdifferentiation, in addition to the destruction of the actomyosin cytoskeleton of fibroblasts in an environment conducive to myogenesis, it is necessary to restore and strengthen the actomyosin cytoskeleton at the right time.

## 4 Experimental Section

### Human and Mouse Fibroblast Cultures

Primary human fibroblasts (HFF‐1y, HFF‐13y, provided by the National Stem Cell Resource Bank, Beijing, China) were established from dissociated foreskin tissue of healthy donors. Human fibroblasts were cultured in fibroblast medium which comprised of Dulbecco's Modified Eagle Medium (DMEM, Gibco, 11995‐065) supplemented with 10% fetal bovine serum (Gibco, 10099141) and 100× penicillin/streptomycin (Gibco, 15140163) at 37 °C in a 5% CO_2_ atmosphere. For cell information, refer to Table S2, Supporting Information.

### Generation of Cd‐iNs

12‐well plates with or without coverslips were coated with 10 µg mL^−1^ poly‐L‐lysine (PDL, Sigma, P6407) overnight, washed with sterile water 3 times, and then coated with 1 µg mL^−1^ laminin (Santa Cruz, sc‐29012) overnight. Fibroblasts were seeded onto Fibronectin‐ or PDL/laminin‐coated plates and cultured in fibroblast medium for 24 h. The cells were transferred into neuronal induction medium with 20 µm (−)‐blebbistatin (Ble, MCE, HY‐13441) or the alternative active derivative, (S)‐(−)‐blebbistatin O‐benzoate (Ble‐OB, TRC, B208070) until cell confluence was about 40–60%. The medium containing Ble was changed every other day. After 7 days, the cells were switched to neuronal maturation medium or optimized maturation medium. Maturation medium was half‐changed every 3 days until the cells were subjected to further analysis.

### N2B27 Medium

Mixing the DMEM/F12 (Gibco, 11320‐033) and Neurobasal (Gibco, 21103‐049) with 1:1 and added with 0.5% N‐2 supplement (Gibco, 17502048), 1% B‐27 supplement (Gibco, 17504044), 10 µg mL^−1^ insulin (Roche Applied Science, 11376497001), *β*‐mercaptoethanol (1000×, Gibco, 21985023), 0.02% bovine serum albumin (BSA, Sigma, A3803), GlutaMAX 100× (Gibco, 10565‐018), and penicillin‐streptomycin.

### Neuronal Induction Medium

N2B27 medium containing 20 ng mL^−1^ recombinant human basic fibroblast growth factor (FGF2, R&D, 233‐FB‐001MG/CF) and 20 µm Ble or Ble‐OB.

### Neuronal Maturation Medium

DMEM/F12 and Neurobasal at a ratio of 1:3, with 0.5% N‐2 (Gibco, 17502048), 1% (NEAA, Gibco, 11140‐050), 1% GlutaMAX (Gibco, 10565‐018), 20 ng mL^−1^ neurotrophins‐3 (NT‐3, PeproTech, 450‐03), 20 ng mL^−1^ brain‐derived neurotrophic factor (PeproTech, 450‐02), 20 ng mL^−1^ glial cell line‐derived neurotrophic factor (*GDNF*, PeproTech, 450‐10), 10 µm Forskolin (Stemgent, 04‐0025), and 10 µm Ble or Ble‐OB. The optimized maturation medium also contained 20–50 µm ISX‐9 (MCE, HY‐12323). The BrainPhys Neuronal Medium N2‐A and SM1 (Stemcell, 05793) replaced the Neurobasal to increase neuronal maturity. Other small molecule inhibitors used in this study are as follows: Y33075 (MCE, HY‐10069), K‐115 (MCE, HY‐15685A), and Verteporfin (MCE, HY‐B0146).

### Immunofluorescence Staining

Immunostaining of cells was performed according to previous report.^[^ ^42^ ^]^ In brief, coverslip cultures were fixed using 4% paraformaldehyde for 15–20 min at room temperature, washed with phosphate buffer saline (PBS, Corning, 21‐040‐CV) 3 times, and then incubated in TBS buffer (contained 2% BSA (Sigma, A3803) and 0.3% Triton X‐100 (Sigma, T8787) in PBS) at room temperature. After 1 h, the primary antibodies (Table S3, Supporting Information) were incubated in TBS buffer overnight at 4 °C. Then, samples were washed with PBS 3 times followed by incubation with the appropriate Alexa‐Fluor‐conjugated secondary antibodies in TBS for 1 h at 37 °C. The nuclei were stained with Hoechst 33342 (Invitrogen, H3570) or DAPI (Sigma, D9542). Images were collected with a laser confocal microscope (ZEISS, LSM 780 META, Germany) or a dual photon laser confocal microscope (Leica, TCS Sp8, Germany). Antibodies used in the study are listed in Table S3, Supporting Information.

### Generation of Neural Fate by siRNA Knockdown

Fibroblasts were seeded onto 10 µg mL^−1^ fibronectin (Millipore, fc010)‐coated plates and cultured in fibroblast medium for 1 day. Then, indicated siRNAs were transfected using RNAi MAX (Life Technologies, 13778) according to the manufacturer's instructions. The siRNA sequences are shown in Table S4, Supporting Information. The medium was changed to N2B27 with 20 ng mL^−1^ FGF2 (Invitrogen, PHG0024) after transfection. The cells were transfected once more after 3 days, then the medium was changed every other day. The cells were stained with the neuron marker TUJ1, the statistics of TUJ1‐expressing cells were counted with Image J. SiRNAs used in the study are listed in Table S4, Supporting Information.

### Quantitative Real‐Time PCR

Purelink RNA Mini Kit was used to extract total RNA (Life Technologies, 12183025) according to the manufacturer's instructions. A total of 2 µg RNA for each reaction was reverse‐transcribed to cDNA with random Primer and M‐MLV Reverse Transcriptase (Promega, M1705). Quantitative real‐time PCR was subsequently conducted with specific primers and 2× SYBR Green Real Time PCR Master Mix (TOYOBO, QPK‐212B) in an MX3000P Stratagene PCR machine. The relative expression levels were normalized with the internal control (GAPDH). Primers used are listed in Table S5, Supporting Information.

### Conversion Efficiency

Conversion efficiency was calculated as previously described.^[^ ^3^ ^]^ Briefly, 10 view fields were randomly selected for each sample. At indicated time points, the total number of TUJ1^+^/MAP2^+^ cells with neuron morphology was counted. The conversion purity was calculated as the ratio of TUJ1^+^/MAP2^+^ hCd‐iNs to the DAPI‐stained cells in each visual field. The conversion efficiency was calculated as the ratio of TUJ1^+^/MAP2^+^ hCd‐iNs to initial cell number. The statistics of indicated positive‐expressing cells were counted with Image J software or Zeiss Zen 2011 light Edition. Data were presented as mean ± SEM from at least three independent experiments.

### Electrophysiology

For electrophysiological recording, Cd‐iNs from eGFP‐labeled HFFs were further co‐cultured with mouse glial cells in neuronal maturation media for 2 weeks. Mouse astrocytes were isolated from brains of neonatal ICR mice and sub‐cultivated for more than 3 passages to eliminate neuron contamination, which was confirmed by examination of mouse Tuj1 protein expression using immunofluorescent staining analysis. Electrophysiological properties were determined by whole cell patch clamp recording at room temperature with external solution (145 mm NaCl, 2.5 mm KCl, 2 mm MgCl_2_, 1 mm CaCl_2_, 10 mm Glucose, 10 mm HEPES, and 0.002 mm Glycine; sodium hydroxide was used to regulate pH to 7.4, 300 mOsm); the patch pipettes were filled with internal solution (10 mm NaCl, 140 mm K‐Gluconate, 1 mm MgCl_2_, 1.1 mm EGTA, and 10 mm HEPES). TTX (10 µm) was used to inhibit the inward sodium currents by perfusion. Potassium hydroxide was used to regulate pH to 7.2, 300 mOsm. The stimulation scheme of the recording voltage‐gated potassium current was Vh = −80 mV and the duration 10 ms; The condition pulse started from −80 mV, and the step 10 mV went to 80 mV, lasted for 200 ms, then recovered to Vh. The stimulation scheme of recording voltage gated sodium current was: Vh = −80 mV and the duration was 10 ms. The condition pulse started at −80 mV, with steps of 10 to 80 mV, lasting for 20 ms, and then recovered to Vh. The stimulus plan recorded the action potential: using the current clamp mode, the maintenance current was 0 pA, starting from 0 pA, 10 mV step, and the interval was recorded at 5 s, which was recorded 20 times.

### Measurement of GABA Release by UPLC‐HRMS

GABA release was measured using a protocol modified from previous report.^[^ ^43^ ^]^ Briefly, the medium was collected with depolarizing Krebs’‐Ringer's buffer (high potassium) containing 50 mm KCl, 83 mm NaCl, 2 mm CaCl_2_, 0.8 mm MgSO_4_, 10 mm glucose, and 20 mm HEPES (pH 7.4) after the cultured cells were washed 4 times with Krebs’‐Ringer's buffer (low potassium) containing 3 mm KCl, 130 mm NaCl, 2 mm CaCl_2_, 0.8 mm MgSO_4_, 10 mm glucose, and 20 mM HEPES (pH 7.4). The collected media (0.5 mL) was mixed with 1 mL borate saline buffer (pH 9.0, 100 mM), subjected to ultrasonic oscillation for 20 min and then the supernatant was centrifuged at 10 000 rpm for 10 min. A 1 mL volume of supernatant was mixed with 500 µL of 3,5‐dinitro‐4‐chloro‐trifluoromethyl‐benzene (100 mM, CNBF, Sigma), an amino compound derivatization reagent for the derivatization reaction.^[^ ^44^ ^]^ Samples were then injected into the ultra‐performance liquid chromatography (UPLC, ACQUITY UPLC I‐Class, Waters, USA) system and analyzed with high resolution mass spectrometry (HRMS, Xevo G2‐S Q‐TOF, Waters, USA). Pure *γ*‐aminobutyric acid (GABA, Sigma, A2129) was used as the standard substance, and different concentrations and the corresponding peak areas were used to construct a standard curve, which was used to calculate the GABA concentration of the sample. Analyses were performed using QuanLynx (Waters, MassLynx4.1, USA).

### RNA‐seq Library Preparation and Data Analysis

Total RNA was isolated from Cd‐iNs at different transdifferentiation stages and human neurons by Trizol (Invitrogen, 15596018). RNA‐seq libraries were prepared with the NEBNextUltra RNA Library Prep Kit for Illumina. Sequencing was performed on a HiSeq X‐Ten sequencer (Illumina, USA) with 150 bp paired‐end sequencing reaction. All RNA‐sequencing data analysis was performed with Hisat2 (version 2.1.0, USA)^[^ ^45^ ^]^ and StringTie (version 2.0.3)^[^ ^46^ ^]^ using the UCSC hg19 annotation in ‐eB mode with default settings. Reads with unique genome locations and genes with no less than 1 fragment per kilobase of exon model per million fragments mapped (FPKM) in at least one sample were used for the next step of analysis. Twofold change of FPKM between two groups was used as the threshold to filter for DEGs. PCA and heatmaps were performed with prcomp and heatmap.2 functions in R. Gene ontology (GO) analysis were performed by DAVID.^[^ ^47^ ^]^ The word cloud showing for GO terms were produced by wordcloud package in R. The annotations for human TFs were downloaded from the TcoF‐DB v2 database.^[^ ^48^ ^]^ The interaction network of differentially expressed TFs were built by STRING online tool STRING: functional protein association networks (https://string‐db.org/) and were determined by Cytoscape.^[^ ^49^ ^]^

### The Single Cell RNA‐seq Library Preparation and Data Analysis

Transdifferentiated single cells at different stages were collected by oral siphon and lysed by cell lysis buffer with RNase inhibitor. RNA‐seq libraries were generated with Smart‐Seq 2 technology performed by Annoroad Gene Technology Co., Ltd, Beijing, China. Sequencing was performed on an Illumina HiSeq X‐Ten in the 2× 150 bp paired‐end mode. The public scRNA‐Seq datasets (GSE67835) for human brain neuron cells were used as controls.^[^ ^27^ ^]^ All data were analyzed in the same pipeline. Briefly, all reads were mapped to human hg19 genome with Hisat2 (version 2.1.0).^[^ ^45^ ^]^ And the gene count matrix was produced by StringTie (version 2.0.3).^[^ ^46^ ^]^ The expression matrix produced in this study and neuron cells from public datasets were first projected into a common space via matrix decomposition. Next, kNN graphs were built for all the cells based on their coordinates in the common space. Neuron cells from public datasets were divided into six clusters by Leiden clustering,^[^ ^50^ ^]^ and cells from the authors’ data were stratified into the different clusters according to the differentiation stage. PAGA2 was then applied to calculate cluster connectivity for all clusters. ForceAtlas^[^ ^51^, ^52^ ^]^ was used for 2D visualization of cells, and the visualization of the cluster connectivity as shown in Figure 2I was generated using PAGA, where thickness of connecting lines represents cluster connectivity. Scanpy 1.4.6,^[^ ^53^ ^]^ the toolkit for single cell data analysis, was used for above analysis.

### Data Availability

The sequencing data have been deposited in Genome Sequence Archive for human (GSA‐Human) of Beijing Institute of Genomics, Chinese Academy of Sciences (https://ngdc.cncb.ac.cn/gsa‐human/). The accession number for the sequencing data reported in this paper is HRA001683.

### Statistical Analysis

All quantitative data were analyzed from at least three independent experiments and are presented as means ± SEM. The Student's *t*‐test was used to calculate statistical significance with *p* values when two groups were analyzed. The *p* values ≤ 0.05 were considered as significantly different, *p ≤ 0.05, ^**^ *p* < 0.01, ^***^ *p* < 0.001, and ^****^ *p* < 0.0001.

## Conflict of Interest

The authors declare no conflict of interest.

## Supporting information

Supporting Information

Supplemental Movie 1

## Data Availability

The data that support the findings of this study are available from the corresponding author upon reasonable request.
