# Supplementary material for: Pharmacological Perturbation of Mechanical Contractility Enables Robust Transdifferentiation of Human Fibroblasts into Neurons
Source: Adv Sci (Weinh). 2022 Mar 3;9(13):2104682. doi: 10.1002/advs.202104682 (PMC9069193; doi:10.1002/advs.202104682)
Supplement: Supplementary file 1 — Supporting Information [file ADVS-9-2104682-s002.pdf]

Supporting Information for

Pharmacological Perturbation of Mechanical Contractility Enables Robust Transdifferentiation of Human Fibroblasts into Neurons

Zheng-Quan He, Yu-Huan Li, Gui-Hai Feng, Xue-Wei Yuan, Zong-Bao Lu, Min Dai, Yan-Ping Hu, Ying Zhang, Qi Zhou, Wei Li

This PDF file includes:

Figs. S1 to S10

Table S1 to S5

Caption for Movie S1

Author Contributions

Figure S1

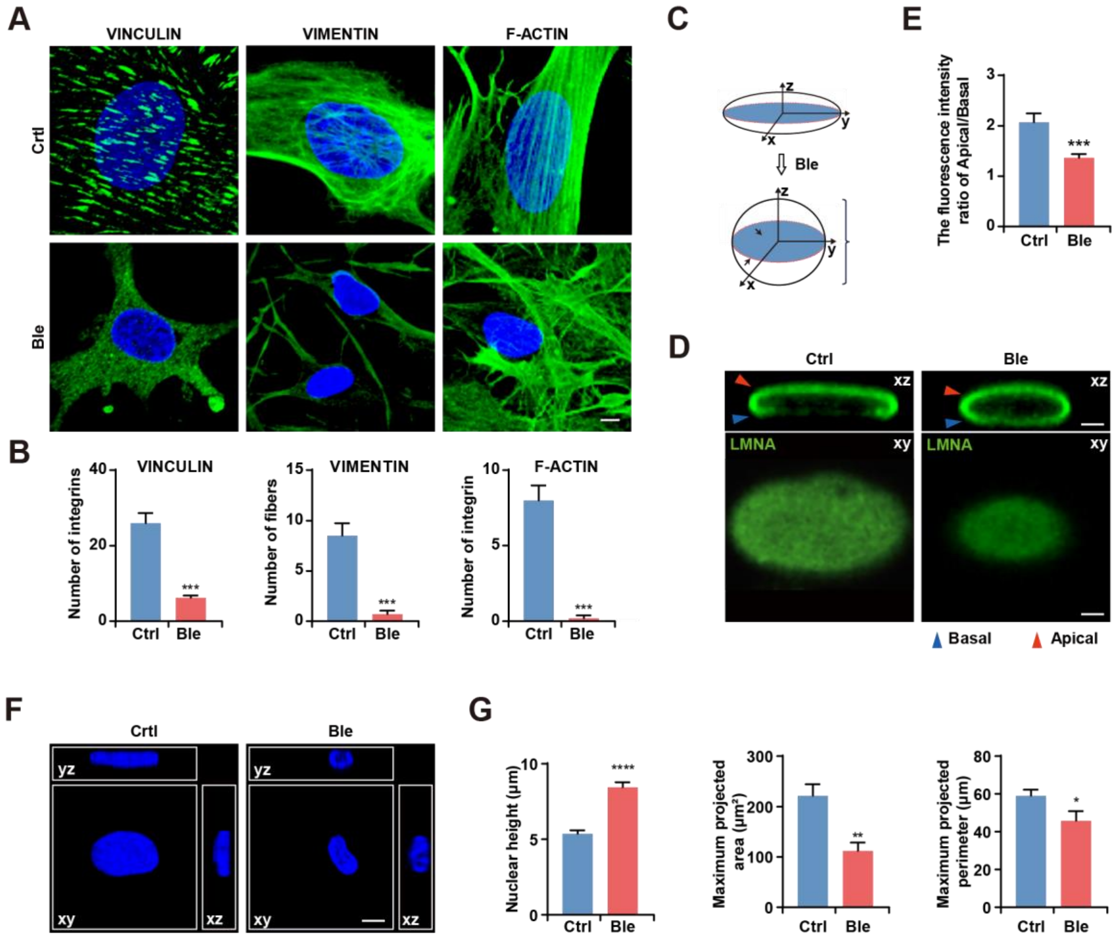

Fig. S1. Ble-mediated inhibition of actomyosin contractility disrupted fibroblast mechanical homeostasis. (A, B) The expression pattern **(A)** and quantification **(B)** of mechanical stress-related proteins, including vinculin, vimentin, and F-actin after Ble treatment. Scale bars, 10 μm. *n*=20 randomly selected cells from 3 repeated experiments. **(C)** 3D diagram of the cell nucleus. **(D, E)** Representative image of nuclear lamina immunofluorescence staining showing reduced apical distribution of LMNA after Ble treatment. Scale bars, 10 μm. *n*=20 randomly selected cells from 3 repeated experiments. **(F, G)** Immunostaining showing the deformed nuclei of human foreskin fibroblasts (HFFs) and the quantitative index (below) after Ble treatment. Scale bars, 20 μm. *n*=50 and 64 randomly selected nuclei from three repeated experiments for Ctrl and Ble treatment. Data represent means ± SEM. *P < 0.05, **P< 0.01, ***P< 0.001 [*t*-test in **(B), (E), (G)**].

Figure S2

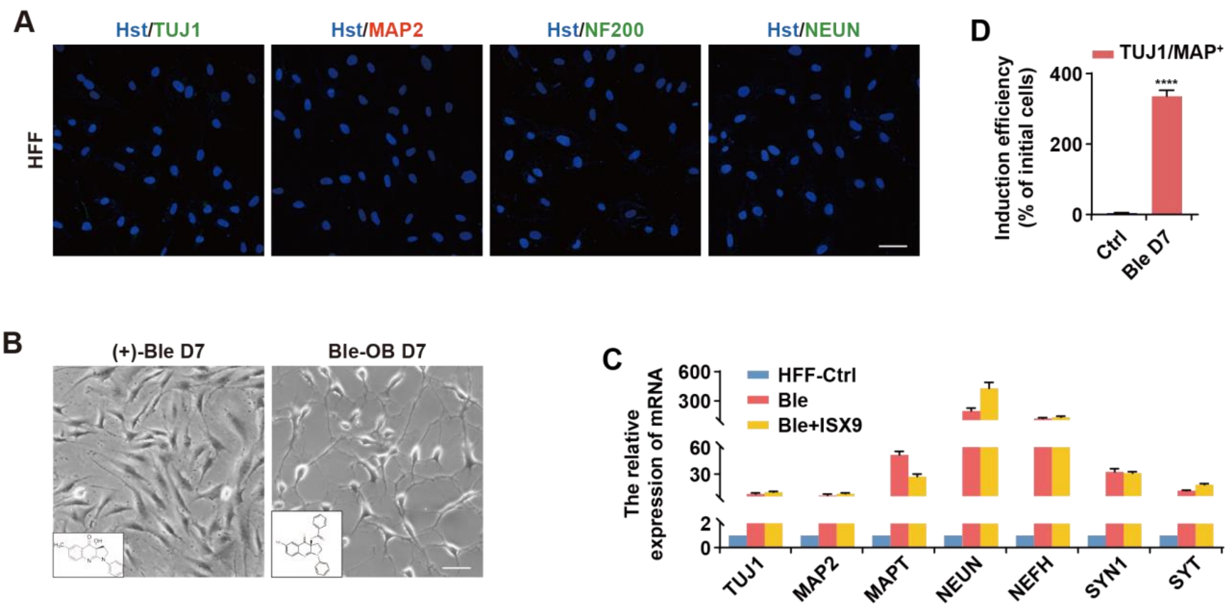

Fig. S2. Ble induced neural trans-differentiation. (A) Ctrl HFFs before Ble treatment are immuno-negative for TUJ1, MAP2, NF200, and NEUN (neuron nuclear antigen) expression. Scale bar, 50 μm. **(B)** Morphology of HFFs after treatment of (+)-Ble ((+)-Blebbistatin) and Ble-OB ((S)-(−)-blebbistatin-O-benzoate) for 7 days. Scale bar, 50 μm. **(C)** qRT-PCR analysis of the expression of TUJ1, MAP2, MAPT, NEUN, NEFH, SYN1, and SYT in HFFs (Ctrl) and cells after Ble and Ble+ISX-9 treatment. ACTIN served as the internal control. *n*=3. **(D)** The efficiency of Fig. 1F. Efficiency = purity * final cell number / initial cell number. *n*=10 randomly selected fields from three repeated experiments.

Figure S3

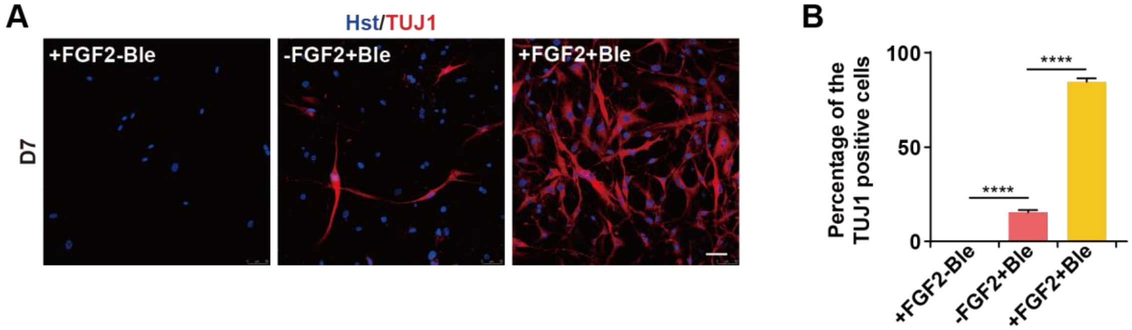

Fig. S3. FGF2 promoted neural trans-differentiation induced by Ble. (A) Immunostaining of TUJ1 (Red) of HFF after 7 days treatment of FGF2 alone, Ble alone, and FGF2 with Ble. Scale bar, 50 μm. **(B)** Quantitation of TUJ1-positive cells relative to Hoechst-stained cells after 7 days treatment of FGF2 alone, Ble alone, and FGF2 with Ble. *n*=3 randomly selected fields from three repeated experiments.

Figure S4

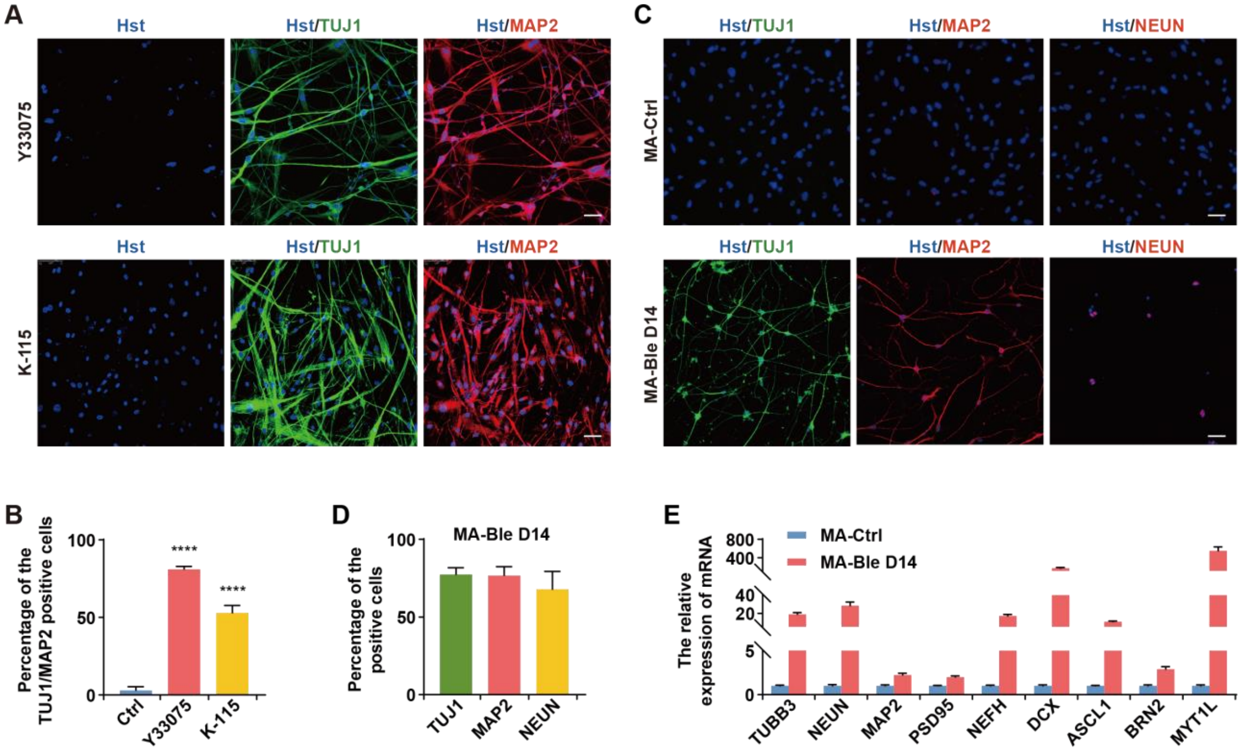

Fig. S4. Inhibition of actomyosin induced different cells neural conversion. (A) HFF-induced neuron-like cells express TUJ1 and MAP2 after Y33075 or K-115 treatment for 14 days. Scale bar, 50 μm. **(B)** Quantitation of TUJ1 or MAP2-positive cells relative to Hst-stained cells after Y33075 or K-115 treatment for 14 days. *n*=10 randomly selected fields from 3 repeat experiments. **(C)** Immunostaining of TUJ1 (green), MAP2 (red), and NEUN (pink) of mouse astrocytes after 14 days of induction and maturation. Scale bar, 50 μm. **(D)** Quantitation of TUJ1, MAP2, NEUN-positive cells relative to Hoechst-stained cells in mouse astrocyte after 14 days treatment of Ble, *n*=10 randomly selected fields from three repeated experiments. **(E)** qRT-PCR analysis of the expression of TUBB3, NEUN, MAP2, PSD95, NEFH, DCX, ASCL1, BRN2, and MYT1L in the primary mouse astrocyte and induced neurons, ACTIN served as the internal control, *n*=3.

Figure S5

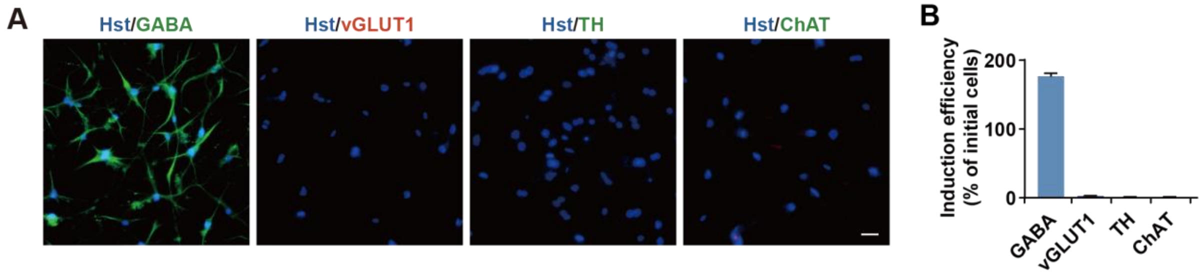

Fig. S5. Identification of neuronal subtypes. (A) Cd-iNs at day 30 were immune-positive for GABA, but immune-negative for vGLUT1, TH, or ChAT. Scale bar, 50 μm. **(B)** The efficiency of Fig. 1O. Efficiency = induced neuron number/initial cell number. *n*=10 randomly selected fields from three repeated experiments.

Figure S6

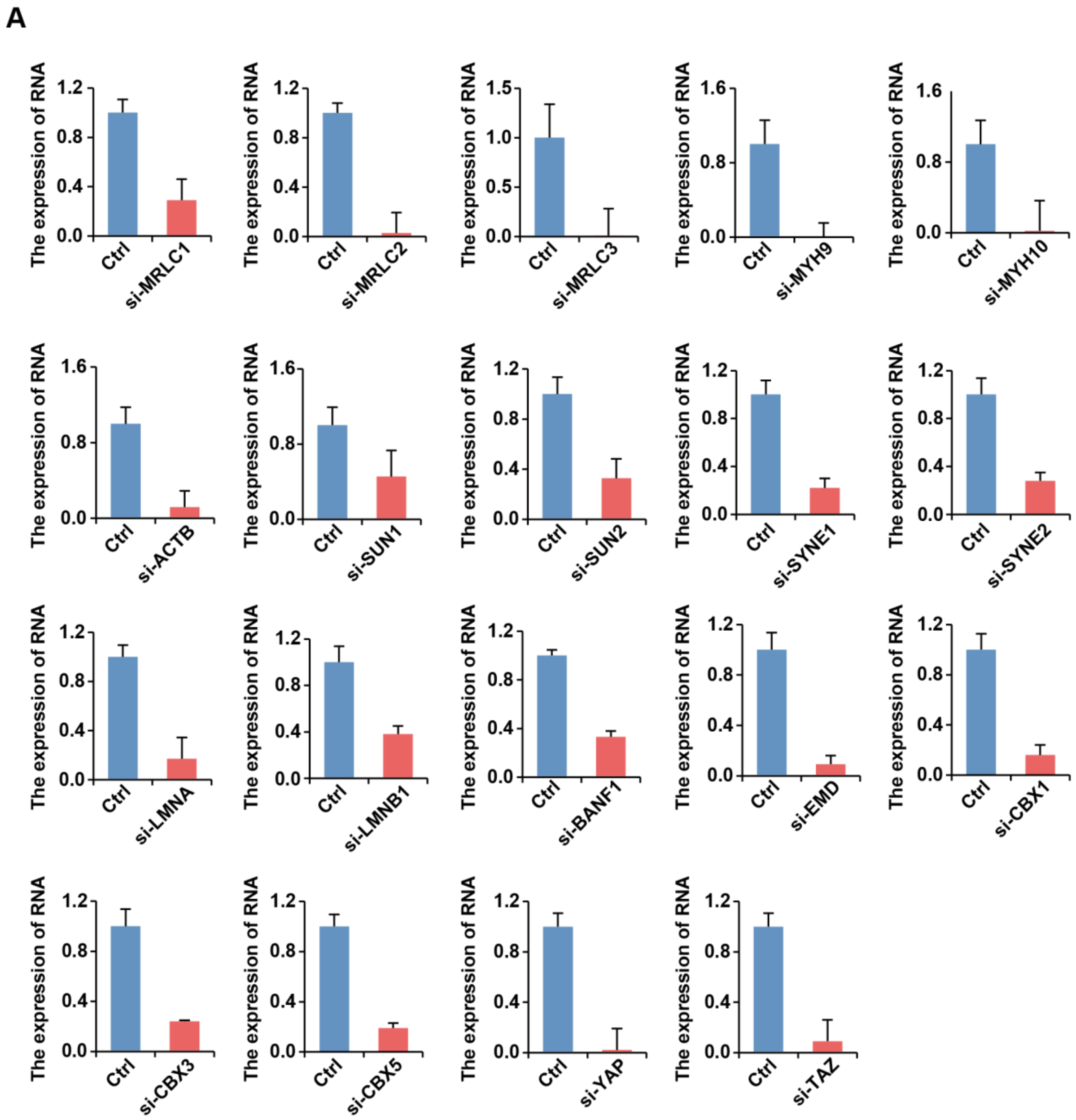

Fig. S6. The effects of siRNAs. Reverse transcription-polymerase chain reaction (RT-PCR) quantification of knockdown efficiency for each siRNA. Data are represented as means ± SEM, *n*=3 repeat experiments.

Figure S7

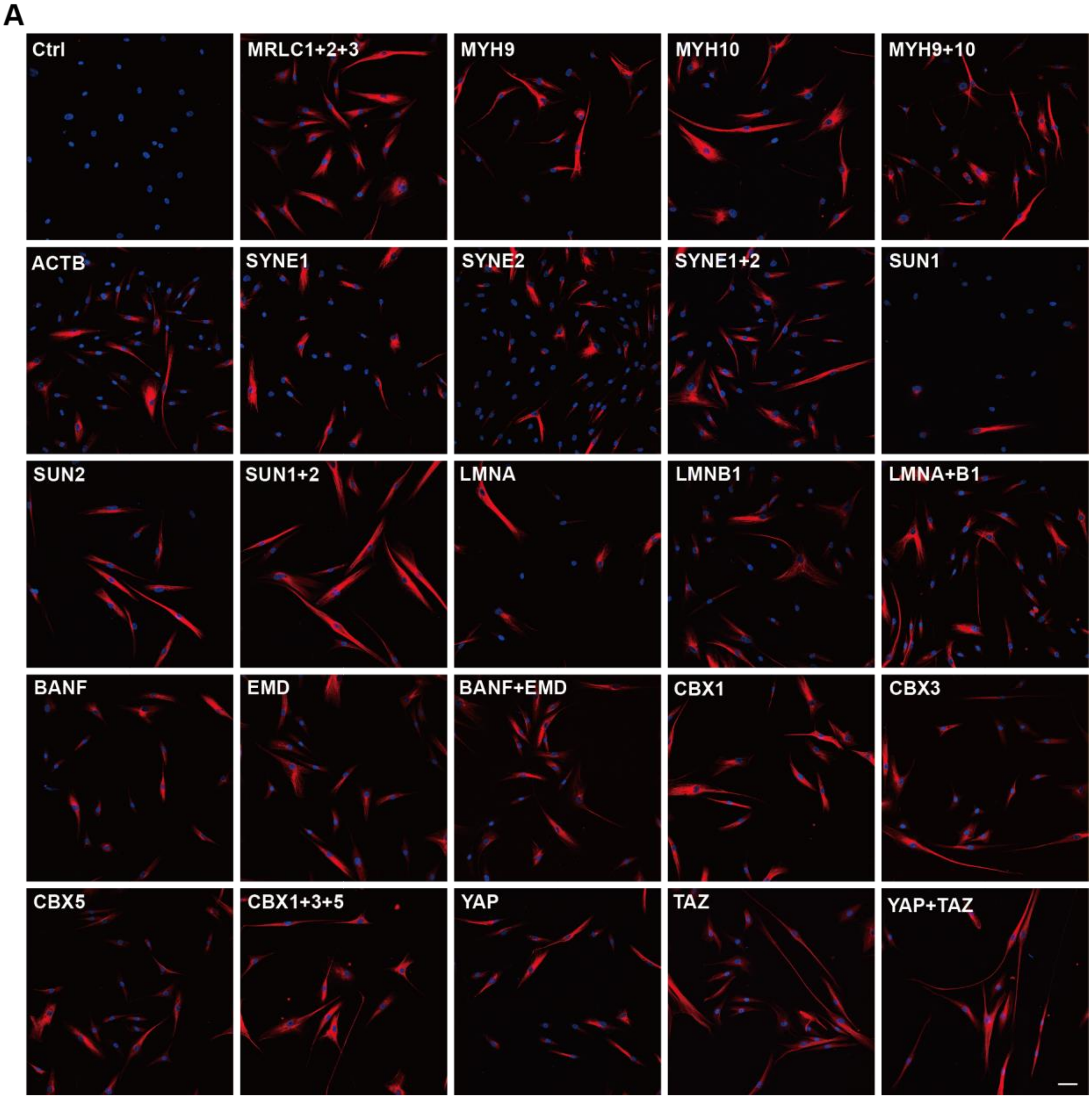

Fig. S7. Representative results of neural-like fate induced by knocking down the cytoskeleton components. (A) Representative of TUJ1-staining after indicated gene knockdown by siRNA treatment. Scale bar, 50 μm.

Figure S8

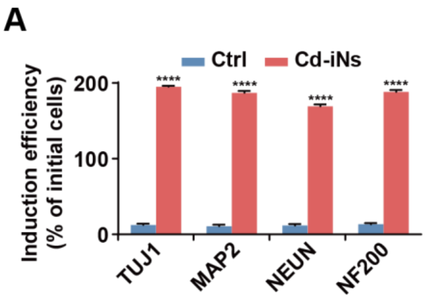

Fig. S8. The efficiency of Cd-iNs in D30. **(A)** Efficiency of **Fig. 2B**. Efficiency = induced neuron number/initial cell number. *n*=10 randomly selected fields from three repeated experiments.

Figure S9

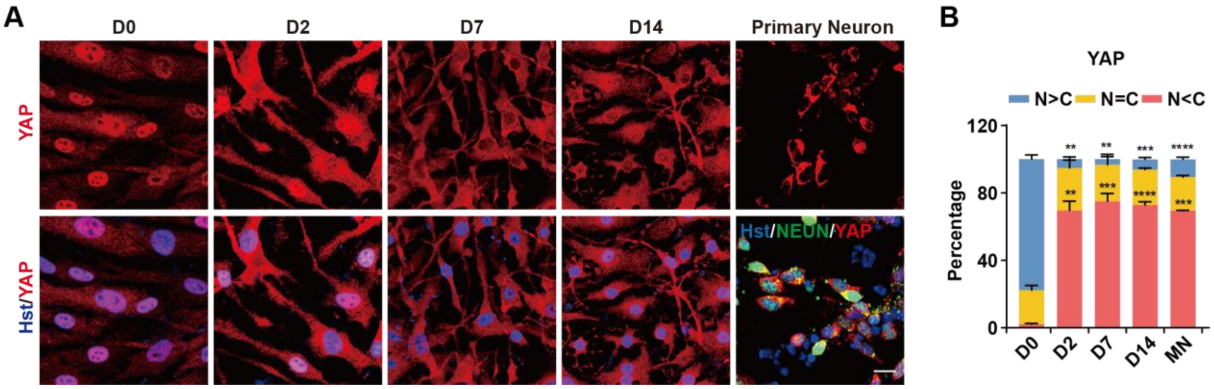

Fig. S9. YAP localization during neural trans-differentiation. (A) Immunostaining of YAP after 0, 2, 7, and 14 days of induction and maturation. Scale bar, 50 μm. **(B)** Statistics for **(A)**. *n*=3 repeated experiments.

Figure S10

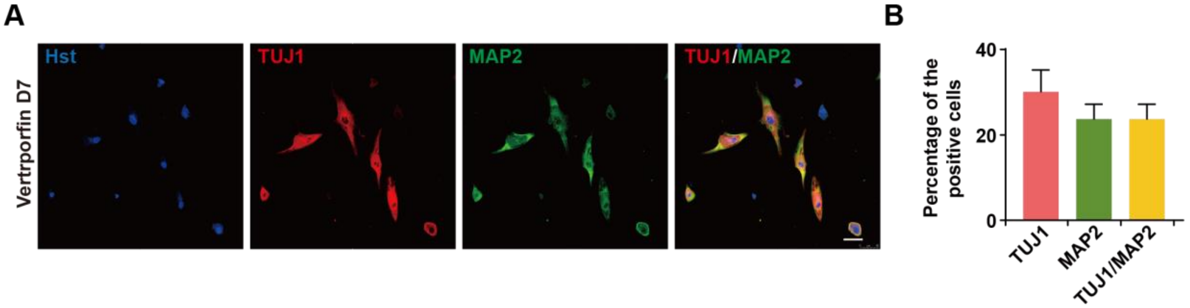

Fig. S10. YAP inhibitor verteporfin promotes neural trans-differentiation. (A) Immunostaining of TUJ1 (red) and MAP2 (green) after 7 days of verteporfin treatment. Scale bar, 50 µm. **(B)** Quantitation of TUJ1 and MAP2-positive cells relative to Hoechst-stained cells after 7 days of verteporfin treatment. *n*=3 randomly selected fields from three repeated experiments.

Table S1. Effects of different siRNAs targeting most cytoskeleton components on neural fate conversion from HFFs.

Table S1

Function	si-Gene	Gene ID	Description	TUJ1^{+}%		Efficiency%	
Actomyosin cytoskeleton and regulatory factors	MRLC1	10398	Myosin light chain 9		18.4±5.1		197.2±54.1
	MRLC2	103910	Myosin light chain 12B				
	MRLC3	10627	Myosin light chain 12A				
	MYH9	4627	Myosin heavy chain 9	13.8±4.5	21.1±3.5	50.1±16.4	86.2±14.5
	MYH10	4628	Myosin heavy chain 10	15.8±3.7		126.1±29.6	
	ACTB	60	Actin beta	3.4±0.2		29.3±2.0	
Linkers of nucleoskeleton and cytoskeleton and heterochromatin	SYNE1	23345	Spectrin repeat containing nuclear envelope protein 1	4.7±1.5	16.3±6.1	14.5±4.7	42.2±15.9
	SYNE2	23224	Spectrin repeat containing nuclear envelope protein 2	8.0±4.1		92.1±47.7	
	SUN1	23353	Sad1 and UNC84 domain containing1	6.9±0.7	32.5±3.6	58.3±6.3	116.3±13.0
	SUN2	25777	Sad1 and UNC84 domain containing2	27.7±4.7		77.2±13.2	
	LMNA	4001	Lamin A/C	9.1±4.3	23.1±2.4	58.0±27.4	288.2±29.0
	LMNB1	4000	Lamin B1	5.0±1.4		63.2±18.3	
	BANF	2010	Barrier to autointegration factor 1	12.9±3.4	14.7±4.7	80.1±20.8	148.9±47.3
	EMD	8815	Emerin	18.4±2.3		233.1±29.1	
	CBX1	10951	Chromobox 1	15.6±2.7	27.4±2.5	104.6±18.3	99.8±9.1
	CBX3	11335	Chromobox 3	15.2±3.2		101.5±21.6	
	CBX5	23468	Chromobox 5	8.9±1.2		102.9±13.4	
Transcriptional regulators in the Hippo pathway	YAP1	10413	Yes associated protein 1	12.9±6.6	24.5±4.3	33.4±17.1	52.7±9.3
	WWTR1	25937	WW domain containing transcription regulator 1	21.1±3.7		131.3±23.0	

Note: Purity = TUJ1-positive cells with circular cell bodies and neurite outgrowth that is at least 3-fold longer than the cell body / Hst-stained cells. Efficiency = induced neuron number / initial cell number.

Table S2

Table of cell lines.

**Cell**	**Passage**	**Cell type**	**Subjects**
HFF-1y	P15-20	Human foreskin fibroblasts	1, male
HFF-13y	P10-15	Human foreskin fibroblasts	13, male
MA	P3-7	Mouse astrocytes	C57-Mice

Table S3

Table of antibodies used in the study.

Antibodies	Source	Identifier
Tubulin β-3 (TUBB3)	Millipore	MAB1637
Tubulin β-3 (TUBB3)	Convance	PRB-435P
MAP2	Santa Cruz	sc-20172
NeuN	Millipore	MAB377
Synaptophysin (SYN1)	Abcam	ab64581
Synaptophysin (SYT1)	Abcam	ab32127
PSD95	Abcam	ab18258
GFP	Abcam	ab13970
vGLUT1	Santa Cruz	sc-377425
TH	Santa Cruz	sc-14007
Choactase	Santa Cruz	sc-20672
GABA	Millipore	MAB316
NF200 (Neurofilament Heavy)	Abcam	ab4680
GAD65/67	Santa Cruz	sc-7513
Calretinin	Abcam	ab702
Parvalbumin	Santa Cruz	sc-7449
Calbindin	Millipore	AB1778
Vinculin	Abcam	ab129002
Vimentin	Santa Cruz	sc-7557
Phalloidin (F-actin)	Cell Signaling Technology	8878S
LAMINA/C	Cell Signaling Technology	4777S
YAP	Santa Cruz	sc-101199

GFAP	Millipore	MAB360

Table S4

Table of siRNAs used in the study

Name	Gene ID	Description	Sequence
MRLC1	10398	Myosin light chain 9	CAAUGUCUUUCGCAAUGUU
MRLC2	103910	Myosin light chain 12B	GCCUCUUCUUUUUGAUGUA
MRLC3	10627	Myosin light chain 12A	GGUCUAUACAGAGUCAAUA
MYH9	4627	Myosin heavy chain 9	GGGUAUCAAUGUGACCGAU
MYH10	4628	Myosin heavy chain 10	GGGCAACUCUACAAAGAAU
ACTB	60	Actin beta	GCATCCACGAAACTACCTT
SUN1	23353	Sad1 and UNC84 domain containing 1	CAGCTTTTAGTATCAACCA
SUN1	25777	Sad1 and UNC84 domain containing 2	GACTCAGAAGACCTCTTCA
SYNE1	23345	Spectrin repeat containing nuclear envelope protein 1	GAACGAGTCTGATTTGATA
SYNE2	23224	Spectrin repeat containing nuclear envelope protein 2	GGTAGAACGTCAACCTCAA
LMNA	4000	Lamin A/C	GAAGGAGGGTGACCTGATA
LMNB1	4001	Lamin B1	CGAGCATCCTCAAGTCGTA
BANF1	8815	Barrier to autointegration factor 1	TGGCCAGTTTCTGGTGCTA
EMD	2010	Emerin	CTCGTAGGCTTTACGAGAA
CBX1	10951	Chromobox 1	GGAAGGGATTCTCAGATGA
CBX3	11335	Chromobox 3	TGACAAACCAAGAGGATTT
CBX5	23468	Chromobox 5	TAAACCCAGGGAGAAGTCA
YAP1	10413	Yes associated protein 1	TCTCTGACCAGAAGATGTC
WWTR1	25937	WW domain containing transcription regulator 1	ACGTTGACTTAGGAACTTT

Table S5

Table of primers used in the study

Primers	Forward	Reverse
hASCL1	5'-TCACCTCTAACACGCACA-3'	5'-AGACGAAAGACACCAACTC-3'
hBRN2	5'-CGGCGGATCAAACTGGGATTT-3'	5'-TTGCGCTGCGATCTTGTCTAT-3'
hTUBB3	5'-CTGGGCCTCCGACTCCTCCTC-3'	5'-CGAGAGCAACATGAACGACCTGGT-3'
hMAP2	5'-TGGTGCCGAGTGAGAAGAAG-3'	5'-AGTGGTTGGTTAATAAGCCGAAG-3'
hNEFH	5'-CCGTCATCAGGCCGACATT-3'	5'-GTTTTCTGTAAGCGGCTATCTCT-3'
hMAPT	5'-GAAGATTGGGTCCCTGGACAATA-3'	5'-AGGTCAGCTTGTGGGTTTCA-3'
hPSD95	5'-AGCTGGAGCAGGAGTTCAC-3'	5'-ACACGCTTCACCTTGTGGTA-3'
hSYN1	5'-AGTTCTTCGGAATGGGGTGAA-3'	5'-CAAACTGCGGTAGTCTCCGTT-3'
hITGAV	5'-ATCTGTGAGGTCGAAACAGGA-3'	5'-TGGAGCATACTCAACAGTCTTTG-3'
hTLN1	5'-GACGATGCAGTTTGAGCCG-3'	5'-GGGTCATCATCTGACAGAAAGAG-3'
hACTIN	5'-CCAACCGCGAGAAGATGA-3'	5'-CCAGAGGCGTACAGGGATAG-3'
hACTN1	5'-TCCATCGGAGCCGAAGAAATC-3'	5'-GTGTCGGTGGATCAAAGCACA-3'
hACTN4	5'-GCAGCATGGGCGACTACAT-3'	5'-TTGAGCCCGTCTCGGAAGT-3'
hFLNA	5'-CTTATCGCGCTGTTGGAGGT-3'	5'-GCCACCGACACGTTCTCAA-3'
hFLNB	5'-GTGAACAAACGCATCGGCAA-3'	5'-ACCAGACCCAAGATGAGCTTC-3'
hFLNC	5'-CTGGGCGATGAGACAGACG-3'	5'-GCGGATGGAACTTGCGGTA-3'
hMRLC1	5'-TCTTCGCAATGTTTGACCAGT-3'	5'-GTTGAAAGCCTCCTTAAACTCCT-3'
hMRLC2	5'-CAGGCACCATTCAGGAAGAT-3'	5'-GTGGGGGAAGTGTCTGAGAA-3'
hMRLC3	5'-TTTTAGCGGCTCTCTGGGTA-3'	5'-CTGAATCTGCGACTGGTCAA-3'
hMYH9	5'-GAAGAGCTAGAGGCGCTGAA-3'	5'-CTTTGCCTTCTCGAGGTTTG-3'
hMYH10	5'-GTACCTTGCCCATGTTGCTT-3'	5'-TTTTGCTTGACGAACAGCAC-3'

hRHOA	5'-AGCCTGTGGAAAGACATGCTT-3'	5'-TCAAACACTGTGGGCACATAC-3'
hRHOC	5'-GGAGGTCTACGTCCCTACTGT-3'	5'-CGCAGTCGATCATAGTCTTCC-3'
hROCK1	5'-AACATGCTGCTGGATAAATCTGG-3'	5'-TGTATCACATCGTACCATGCCT-3'
hROCK2	5'-TGAAGCCTGACAACATGCTC-3'	5'-TCTCGCCCATAGAAACCATC-3'
hMRCKα	5'-GGTGATTGGTCGAGGAGCTTT-3'	5'-TCACGAAAACATGCTGTCTCAG-3'
hMRCKβ	5'-CACTACGCCTTTCAGGACGAG-3'	5'-GAGCAGGGTCAGTAAATCACC-3'
hMYLK	5'-CCCGAGGTTGTCTGGTTCAAA-3'	5'-GCAGGTGTACTTGGCATCGT-3'
hLBR	5'-CGAGGGAGTCGATCAAGGTCA-3'	5'-CTTCAGAATCAGCGGAGTCAAT-3'
hSUN1	5'-ATGTCCCGCCGTAGTTTGC-3'	5'-CCGTCGAGTCACAGCATCC-3'
hSUN2	5'-TGACGTGCCTGACGTATGG-3'	5'-AAATGTGGCGATGAGTCTCTG-3'
hLMNA/C	5'-AATGATCGCTTGGCGGTCTAC-3'	5'-CACCTCTTCAGACTCGGTGAT-3'
hLMNB1	5'-AAGCATGAAACGCGCTTGG-3'	5'-AGTTTGGCATGGTAAGTCTGC-3'
hLMNB2	5'-GTCCTGGATGAGACGGCTC-3'	5'-GCGCTCTTGTTGACCTCGT-3'
hCBX1	5'-GCCGGAGCGGATTATTGGAG-3'	5'-GTGGGCACTTGACATTGGC-3'
hCBX3	5'-TAGATCGACGTGTAGTGAATGGG-3'	5'-TGTCTGTGGCACCAATTATTCTT-3'
hCBX5	5'-TTGCCCTGAGCTAATTTCTGAAT-3'	5'-GATGTCATCGGCACTGTTTGA-3'
hYAP1	5'-TAGCCCTGCGTAGCCAGTTA-3'	5'-TCATGCTTAGTCCACTGTCTGT-3'
hCYR61	5'-CTCGCCTTAGTCGTCACCC-3'	5'-CGCCGAAGTTGCATTCCAG-3'
hCTGF	5'-CAGCATGGACGTTCGTCTG-3'	5'-AACCACGGTTTGGTCCTTGG-3'
hBIRC5	5'-GGCCCAGTGTTTCTTCTGCTT-3'	5'-GCAACCGGACGAATGCTTT-3'
hSYNE1	5'-ACCTCCAATGGTGGTGGAC-3'	5'-CGTGCCAATGTTAGCCACA-3'
hSYNE2	5'-ACCACCCTATGGAAAGCTACT-3'	5'-CATCTCCCATCTGTCGAAGGC-3'
hEMD	5'-GCAGCTACCAAAACGTCTATGT-3'	5'-CACCTTGAAGAAGTGTGTGGG-3'
hBANF1	5'-TGGCTGAAAGACACTTGTGG-3'	5'-CACTCTCGAAGGCATCCGAAG-3'
hWWTR1	5'-TCCCAGCCAAATCTCGTGATG-3'	5'-AGCGCATTGGGCATACTCAT-3'

hGAPDH	5'-ACCACAGTCCATGCCATCAC-3'	5'-TCCACCACCCTGTTGCTGTA-3'
mTUBB3	5'-TAGACCCCAGCGGCAACTAT-3'	5'-GTTCCAGGTTCCAAGTCCACC-3'
mNEUN	5'-ATCGTAGAGGGACGGAAAATTGA-3'	5'-GTTCCCAGGCTTCTTATTGGTC-3'
mMAP2	5'-GCCAGCCTCAGAACAAACAG-3'	5'-AAGGTCTTGGGAGGGAAGAAC-3'
mPSD95	5'-TGAGATCAGTCATAGCAGCTACT-3'	5'-CTTCCTCCCCTAGCAGGTCC-3'
mNEFH	5'-AGACCCCCGTCAAGGAAGG-3'	5'-CTTCTCAGGGGATTTCGCCT-3'
mDCX	5'-CATTTTGACGAACGAGACAAAGC-3'	5'-TGGAAGTCCATTCATCCGTGA-3'
mASCL1	5'-GCAACCGGGTCAAGTTGGT-3'	5'-GTCGTTGGAGTAGTTGGGGG-3'
mBRN2	5'-AGCAGTTCGCTAAGCAGTTCA-3'	5'-CGAAGCGGCAGATAGTGGTC-3'
mMYT1L	5'-TGGTCACGTCAGTGGCAAATA-3'	5'-TGCAAATGGTTTTCGCTTGGG-3'

Movie S1

Ble induces rapid morphological changes of HFFs.

Author contributions

W. Li and Q. Zhou conceived and designed the experiments; Z. Q. He and Y. H. Li performed the induction and identification experiments; G. H. Feng and M. Dai analyzed the bulk and single-cell RNA sequencing data; Z. Q. He, Z. B. Lu and X. W. Yuan performed YAP/TAZ related experiments; Z. Q. He and Y. H. Li performed the other experiments and analyses with the help of X. W. Yuan, Z. B. Lu, and Y. P. Hu. Y. Zhang provided insightful suggestions for the preparation of the manuscript. Z. H. He and W. Li wrote the manuscript with the help of the other authors.
